# PSR: polymorphic SSR retrieval

**DOI:** 10.1186/s13104-015-1474-4

**Published:** 2015-10-01

**Authors:** Concita Cantarella, Nunzio D’Agostino

**Affiliations:** Consiglio per la ricerca in agricoltura e l’analisi dell’economia agraria - Centro di ricerca per l’orticoltura, Via Cavalleggeri 25, 84098 Pontecagnano Faiano, Italy

**Keywords:** Simple sequence repeats, Length polymorphism, Polymorphic microsatellites, NGS, SAM/BAM format

## Abstract

**Background:**

With the advent of high-throughput sequencing technologies large-scale identification of microsatellites became affordable and was especially directed to non-model species. By contrast, few efforts have been published toward the automatic identification of polymorphic microsatellites by exploiting sequence redundancy. Few tools for genotyping microsatellite repeats have been implemented so far that are able to manage huge amount of sequence data and handle the SAM/BAM file format. Most of them have been developed for and tested on human or model organisms with high quality reference genomes.

**Results:**

In this note we describe *polymorphic SSR retrieval* (*PSR*), a read counter and simple sequence repeat (SSR) length polymorphism detection tool. It is written in Perl and was developed to identify length polymorphisms in perfect microsatellites exploiting next generation sequencing (NGS) data. *PSR* has been developed bearing in mind plant non-model species for which *de novo* transcriptome assembly is generally the first sequence resource available to be used for SSR-mining. *PSR* is divided into two modules: the read-counting module (*PSR_read_retrieval*) identifies all the reads that cover the full-length of perfect microsatellites; the comparative module (*PSR_poly_finder*) detects both heterozygous and homozygous alleles at each microsatellite *locus* across all genotypes under investigation. Two threshold values to call a length polymorphism and reduce the number of false positives can be defined by the user: the minimum number of reads overlapping the repetitive stretch and the minimum read depth. The first parameter determines if the microsatellite-containing sequence must be processed or not, while the second one is decisive for the identification of minor alleles. *PSR* was tested on two different case studies. The first study aims at the identification of polymorphic SSRs in a set of *de novo* assembled transcripts defined by RNA-sequencing of two different plant genotypes. The second research activity aims to investigate sequence variations within a collection of newly sequenced chloroplast genomes. In both the cases PSR results are in agreement with those obtained by capillary gel separation.

**Conclusion:**

*PSR* has been specifically developed from the need to automate the gene-based and genome-wide identification of polymorphic microsatellites from NGS data. It overcomes the limits related to the existing and time-consuming efforts based on tools developed in the pre-NGS era.

## Background

Simple sequence repeat (SSR), also known as short tandem repeats (STRs), variable number tandem repeats (VNTRs) or microsatellite markers, are repetitive stretches of 1–6 nucleotide units randomly spread in eukaryotic genomes. Microsatellites typically vary in length between 5 and 40 repeat units and can be classified as perfect or imperfect: in the first case, the repeat unit is arranged in head to tail manner without any interruption; in the second case the repeat unit includes insertions, deletions or substitutions of bases. In addition, SSRs can be of two types: simple or compound (i.e. two adjacent distinct SSRs are separated by a certain number of nucleotides). A further categorization allows SSRs to be recognized as genomic SSRs (gSSRs) and expressed sequence tag (EST)-SSRs; the latter result from transcribed regions of a genome, are associated with functionally annotated genes and are supposed to be more conserved among related species/genera. SSRs are very polymorphic due to the high mutation rate affecting the number of repeat units and have several advantages over other molecular markers: they allow the identification of multiple alleles at single *locus*, are equally distributed all over the genome, show a co-dominant inheritance pattern and could be mapped in different populations becoming the “backbone” of high resolution linkage maps. The development of EST-SSR markers was historically based on collection of sequence data of complementary DNAs (cDNAs) generated using the Sanger sequencing technology. Some useful tools have been made available for the detection and localization of both perfect and imperfect microsatellites along genome and mRNA sequences [1–6] (http://www.bioinformatics.org/ftp/pub/msatfinder/; http://pgrc.ipk-gatersleben.de/misa/).

Generally, these tools are equipped with additional programs able to design primers for microsatellite flanking regions. One of the most common approaches to the detection of length polymorphisms relies on the random selection of SSRs and on the analysis of PCR products on high-resolution gels [7]. Since the application of this method is labour-intensive and time-consuming, it is more advisable to adopt an in silico approach to identify polymorphic SSRs and subsequently validate them through capillary gel separation. Relatively, little work has been done till now toward the automatic identification of polymorphic SSRs by exploiting EST sequence redundancy [4]. With the advent of next-generation sequencing (NGS) methods, the large-scale identification of microsatellites became rapid and cost-effective [8] and was especially directed to non-model species with un-sequenced genomes [9–14]. But, high-throughput NGS technologies posed new challenges to existing bioinformatics applications that need to face a huge amount of sequence data and to handle new file formats. Until a few years ago, no adequate tools have been developed for the identification of polymorphic SSRs from NGS data with the exception of custom scripts still based on the detection of pad characters in the output of the final CAP3 assembly [9] or of the use of graphical viewer for NGS assemblies as starting point to manually explore and estimate microsatellite variability at each SSR *locus* [12, 15, 16]. It is clear, therefore, that it is no longer possible to work with human-readable text files including multiple sequence alignments of reads to the reference; rather it is essential to consider compressed text files in SAM/BAM format [17] to analyse sequence alignments and extract useful patterns. As far as we know, lobSTR [18], RepeatSeq [19] and the recently developed STR-FM [20] are the mainly publically available tools for profiling microsatellites from SAM/BAM data. All of them were conceived to identify gSSR alleles at each *locus* bearing in mind the short size of NGS reads and the difficulties this type of sequence data arises in the correct identification of polymorphic SSRs. An additional effort has been freshly published [15], but the method described is not publically available. However, these tools, even though capable of genotyping also EST-SSRs, were developed for and tested on human or model organisms for which the genome is available and represents a good reference for SSR-mining. Indeed, it seems that their adaptability is not likely to allow the easy use of custom reference transcriptomes and/or automatic comparative analysis at microsatellite *loci*. In this paper, we present *polymorphic SSR retrieval* (*PSR*) a Perl package developed to identify polymorphic SSRs from NGS data and provide quantitative information to each call. Unlike the aforementioned tools, *PSR* has been developed in a context where *de novo* transcriptome assembly is generally the first sequence resource for non-model plant species to be used for SSR-mining and downstream investigations.

## PSR workflow

*PSR* is a Perl package, conceived as a modular and flexible tool, developed to identify polymorphic SSRs from NGS data. The *PSR* package and the user manual are available at the following URL: http://sourceforge.net/projects/polyssr/.

**Fig. 1:**
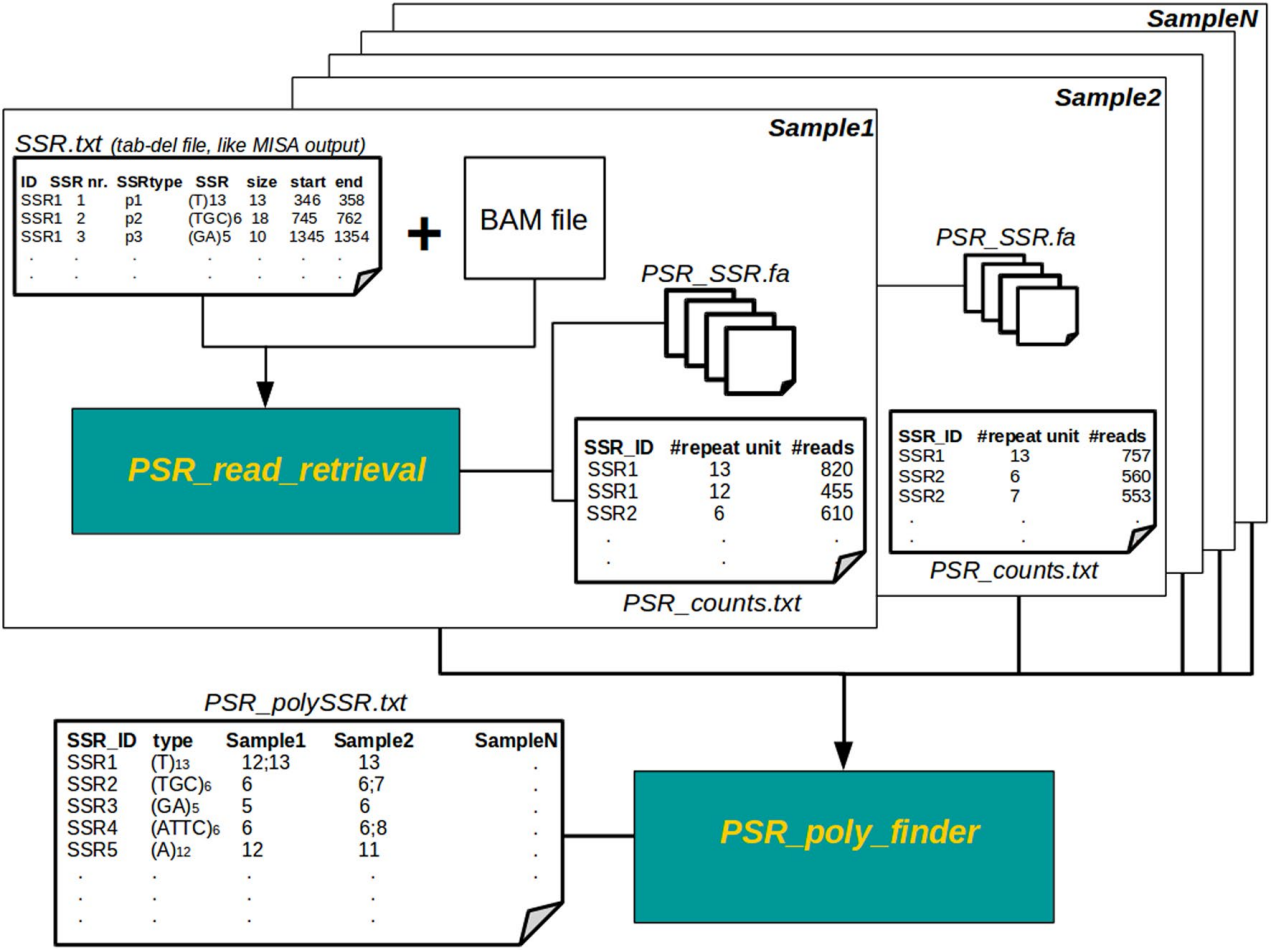
Workflow design of the polymorphic SSR retrieval tool that includes two modules. *PSR_read_retrieval* aims at the identification of all the reads that cover the full-length of perfect microsatellites. N indicates the number of iteration that must correspond to the number of genotypes under investigation. *PSR_poly_finder* detects length polymorphism in microsatellites

**Fig. 2:**
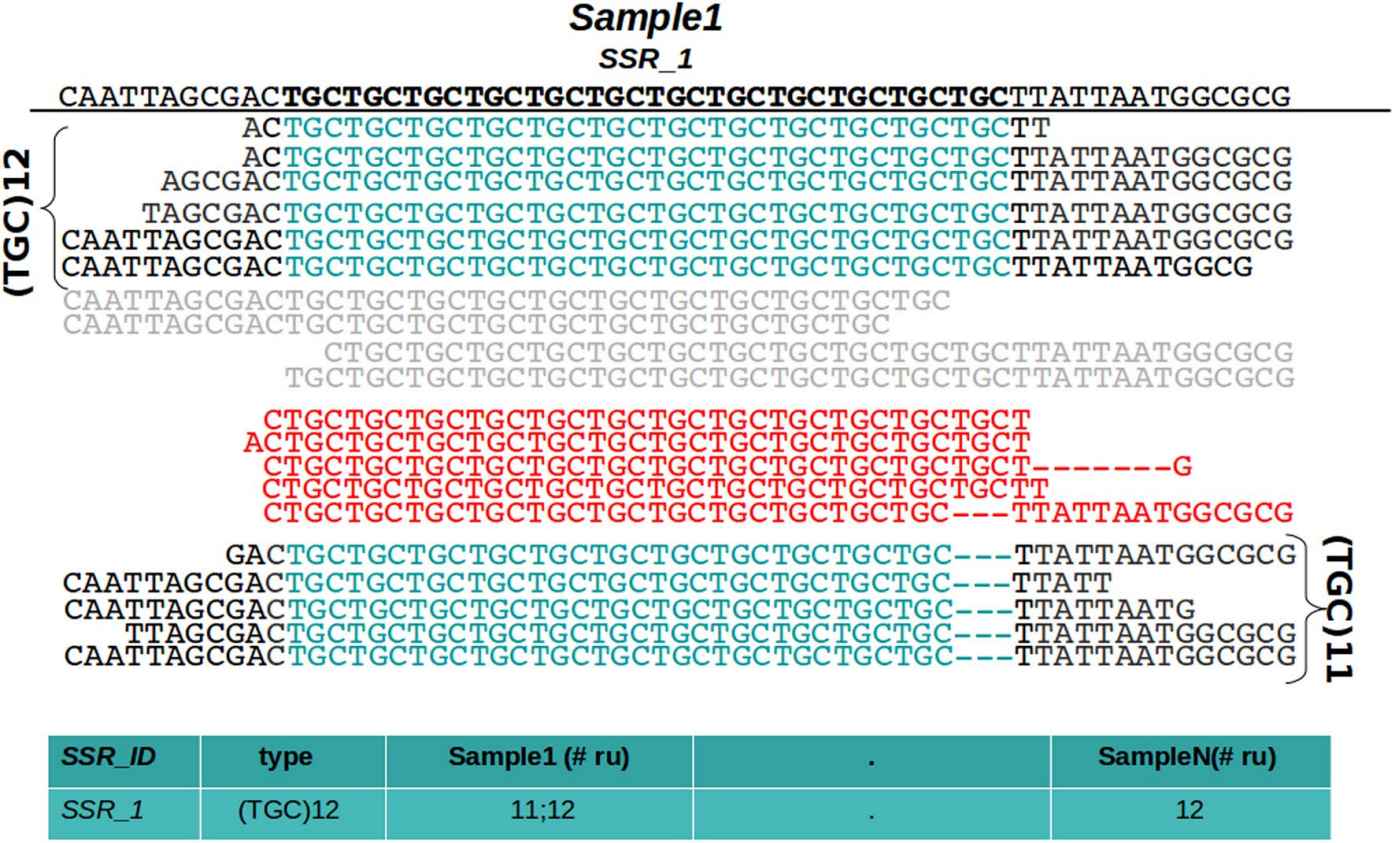
The microsatellite region (36 nucleotides in length) in the reference sequence is shown in *bold*. *PSR* selects and counts only reads aligning the entire SSR region, in addition to one or more nucleotides to the *left* and *right side* different from bases in the repetitive units. Reads in *grey* are discarded since they partially cover the microsatellite. Reads in *red* are filtered out due to the uncertainty at the sequence borders. For each sample the program returns the number of repetitive units presenting coverage greater than the selected threshold (−p) in a tab delimited file

The user guide provides general information on software dependencies and installation procedure as well as detailed instructions for running the application. *PSR* workflow is shown in Fig. 1 and the text that follows describes the key points. *Psr_read_retrieval* aims at the identification of all the reads that align to the reference sequences covering the full-length of perfect microsatellites. We decided to focus on perfect microsatellite only, because the total length of the reads obtained by the Illumina sequencing instruments rarely exceeds the 100 high quality nucleotides. These constraints appear to limit polymorphism discovery only to short polymorphic SSRs, but as it is evident from data in Table 1, the maximum length of the microsatellite is always slightly lower than the one of the sequenced read. In addition, the number of reads covering each microsatellite does not reflect the total number of reads at each *locus*. Indeed, in case SSRs are located at the ends of the read, the microsatellite-containing sequences are discarded since they can strongly affect the call of polymorphic sites (Fig. 2).

**Table 1:** The average read length obtained from Illumina instruments is typically less than 150 nucleotides

Repeat unit (nts)	Max number of repeat unit	SSR length (nts)
Read length = 100 nucleotides (nts)		
2	48	96
3	31	94
4	23	92
5	18	90
6	14	88

This length allows to detect di-nucleotide repetition up to 96 nucleotides and 14 repetitions of esa-nucleotide pattern

In addition, *PSR* filters out all the reads that match twice or more on the reference sequence as well as non-overlapping paired-end reads that aligned on the same microsatellite. This filtering step allows the user to exclude misalignments that can greatly influence downstream analysis.

Sometimes it could happen that the most frequent microsatellite identified by *PSR* presents a number of repeat units that differs from the one detected on the microsatellite-containing reference sequence. This is not surprising since the reference sequence was assembled considering all the reads; by contrast *PSR* takes into account only those reads that entirely overlap the repeating pattern.

*Psr_read_retrieval* takes two files as input: a 7-column tab-delimited file and a BAM file. The first one is a MISA-like output file (http://pgrc.ipk-gatersleben.de/misa/misa.html) that includes the type and the size of identified microsatellites and their localization within the reference sequences. The second input file stores read alignments against reference sequences for each genotype under investigation. The latter can be genome sequences or *de novo* assembled transcripts depending on the type of microsatellites under study.

Based on our experience and on results by Highnam et al. [19], we suggest bowtie2 [21] as reference sequence alignment mapping tool. However, we are well-aware that read mapping tools are constantly being developed; for that reason we believe more convenient to stay independent from alignment algorithm.

*Psr_read_retrieval* should be run as many times as the number of genotypes under investigation (N in Fig. 1). It generates two different outputs: a collection of FASTA files including all the reads that successfully aligned along each SSR and different 3-column tab-delimited files, one for each genotype under investigation, where for each microsatellite the number of repeat units and the number of supporting reads are reported. In case of genomic DNA, results from this step of the analysis allowed ambiguities associated with repetitive stretches to be fixed as well as sequence assembly quality to be assessed.

Then, all the table-wise text files are fed into the *psr_poly_finder* module that, exploiting MySQL DBMS, creates a query table joining all the info recorded in text files in order to detect both heterozygous and homozygous alleles at each microsatellite *locus*. Users can define two different parameters, namely the minimum number of supporting reads (−t) and the minimum read depth (−p), for length polymorphism to be called. By default the values of these parameters are fixed to 10 and 30, respectively.

To give a clear example, a microsatellite is analysed in case at least ten reads overlap the repetitive stretch and it is called as heterozygous if 30 % or more of the reads supports the identification of the minor allele. In case of polyploid genotypes, the user can fine-tune the value of the “p” parameter in order to support the detection of all the putative alleles at each *locus*.

Filtering by a minimum number of reads supports variant calls and by minimum read depth within the microsatellite interval helps to reduce false positive calls.

*Psr_poly_finder* produces as final output a table-wise text file where the first two columns report the identifiers of microsatellite-containing sequences and the type of the identified microsatellite; the remaining columns include one or more (in case of heterozygosity) SSR allele sizes per genotype.

In order to evaluate *PSR* reliability and effectiveness, the package has been tested in the identification of polymorphic SSRs in (1) a collection of *de novo* assembled transcripts defined by RNA-sequencing of two different plant genotypes; (2) a set of newly sequenced chloroplast genomes.

In the first case, ten randomly selected polymorphic microsatellites, as they were identified by *PSR,* were assessed by capillary electrophoresis and eight of them showed the expected length polymorphism (Table 2). Only one SSR *locus* is heterozygous in case of genotype 1 and results from in silico and wet-lab experiments are in agreement. As for genotype 2, seven out of ten microsatellite *loci* resulted heterozygous even if *PSR* identifies both the alleles (major and minor) only in five cases.

**Table 2:** Evaluation through capillary gel separation of SSR length polymorphism from ten randomly selected transcripts

Seq ID	SSR	Start	Stop	SSR length	SSR type	Capillary electrophoresis		PSR	
						Genotype 1	Genotype 2	Genotype 1	Genotype 2
TR11073	(GAT)6	389	406	18	p3	222	222 + 219	6	6; 5
TR11727	(TCA)5	1026	1040	15	p3	182	179	6	5
TR12365	(GAT)6	269	286	18	p3	243 + 246	243 + 246	8; 6	8; 6
TR19012	(CAC)7	208	228	21	p3	239	233 + 239	7	7; 5
TR6251	(CAA)9	204	230	27	p3	294	294 + 297	9	10; 9
TR1824	(CAT)5	90	104	15	p3	277	274	6	5
TR12469	(TC)7	1300	1313	14	p2	234	232	7	6
TR7469	(ATC)7	20	40	21	p3	237	237 + 240	7	7; 8
TR2455	(TTG)6	57	74	18	p3	239	239 + 242	6	6
TR142	(TCT)9	1874	1900	27	p3	159	156 + 159	9	9

Columns 7 and 8 report amplicon size detected into two genotypes. Columns 9 and 10 list the number of repeated units as identified by PSR

In the latter case *PSR* combined with capillary gel separation was used to assess whether in silico identified length polymorphisms represented sequencing/assembling errors or, alternatively, real genotype-specific sequence variations. In seven cases out of eight, the size of the microsatellite supported by the highest number of reads was confirmed by capillary electrophoresis (Table 3). The use of *PSR* proved to be particularly useful to fix sequencing errors in monomeric microsatellites.

**Table 3:** Evaluation through capillary electrophoresis of eight monomeric SSR *loci* across nine cpDNA genotypes

Sample	SSR1 (T)n	SSR2 (T)n	SSR3 (T)n	SSR4 (A)n	SSR5 (T)n	SSR6 (A)n	SSR7 (A)n	SSR8 (A)n
G1	*11*	*13*	*16*	*12*	*16*	*9*	*16*	*18*
G2	*13*	13	16	12	*13*	***9***	13	*16*
G3	12	13	16	13	*17*	***10***	16	15
G4	*12*	13	16	*13*	17	***10***	16	*15*
G5	12	13	16	13	17	***10***	16	15
G6	12	13	16	13	17	***10***	16	15
G7	12	13	16	13	17	***10***	16	15
G8	12	13	16	13	17	***10***	16	15
G9	12	13	16	13	17	***10***	15	15

Numbers represent the length of SSR stretches. Italics cells indicate microsatellites that have been confirmed also by Sanger sequencing. Bold italics cells represent SSRs with different lengths compared to those determined by *PSR*

It is very likely that differences from in silico and wet-lab experiments depend on the amount of reads *PSR* uses to determine the number of repeated unit and microsatellite length. Indeed, for each SSR region *PSR* discards all the reads (on average the 60 % of the total) that do not completely span the microsatellite. Furthermore, in case of EST-SSRs, the quality of the reference transcriptome may negatively affect read alignment as a first step and, subsequently, read count. Finally, differences can be the result of PCR slippage products of repetitive stretches or can be ascribed to errors generated by PCR-based NGS technologies, especially in case of monomeric microsatellites [20].

## Conclusions

The ability of next generation sequencing technologies to produce large amounts of sequence data accelerated microsatellite identification and facilitated the discovery of polymorphic SSRs. In addition, NGS resulted in the development of new specialised tools and file formats that support the management of huge amount of sequence data. However, as far as we know, few automatic and efficient systems have been developed so far for detecting both gene-based and genome-wide polymorphic microsatellites from NGS data. In this note we proposed *PSR*, a Perl Package developed for the detection of length polymorphisms and for the automatic comparative analysis at microsatellite *loci.* Indeed, the availability of polymorphic *SSR* markers is an essential requirement for plant community since it serve as basic resources for QTL mapping, molecular breeding, genetic diversity analysis and SSR fingerprinting for food traceability. All these and further applications, motivated us to develop and distribute *PSR* within plant community.

## Availability and requirements

- Project name: PSR—polymorphic SSR retrieval
- Project home page: http://sourceforge.net/projects/polyssr/
- Operating system(s): tested on Linux and Mac OS X
- Programming language: Perl
- Other requirements: BioPerl, MySQL
- License: GNU GPL 3
- Restrictions to use by non-academics: None.

## Abbreviations

PSR: polymorphic SSR retrieval
SSR: simple sequence repeat
STR: short tandem repeat
VNTR: variable number tandem repeats
gSSRs: genomic SSRs
EST-SSRs: expressed sequence tag-SSR
NGS: next generation sequencing
QTL: quantitative trait *loci*
DBMS: database management system

## References

[1] Benson G (1999). Tandem repeats finder: a program to analyze DNA sequences. Nucleic Acids Res.

[2] Kolpakov R, Bana G, Kucherov G (2003). mreps: efficient and flexible detection of tandem repeats in DNA. Nucleic Acids Res.

[3] da Maia LC, Palmieri DA, de Souza VQ, Kopp MM, de Carvalho FI, Costa de Oliveira A (2008). SSR locator: tool for simple sequence repeat discovery integrated with primer design and PCR simulation. Int J Plant Genom..

[4] Tang J, Baldwin SJ, Jacobs JM, Linden CG, Voorrips RE, Leunissen JA (2008). Large-scale identification of polymorphic microsatellites using an in silico approach. BMC Bioinform.

[5] Temnykh S, DeClerck G, Lukashova A, Lipovich L, Cartinhour S, McCouch S (2001). Computational and experimental analysis of microsatellites in rice (*Oryza sativa* L.): frequency, length variation, transposon associations, and genetic marker potential. Genome Res.

[6] Churbanov A, Ryan R, Hasan N, Bailey D, Chen H, Milligan B (2012). HighSSR: high-throughput SSR characterization and locus development from next-gen sequencing data. Bioinformatics.

[7] Chandra A, Grisham MP, Pan YB (2014). Allelic divergence and cultivar-specific SSR alleles revealed by capillary electrophoresis using fluorescence-labeled SSR markers in sugarcane. Genome/Natl Res Counc Can = Genome/Conseil national de recherches Can..

[8] Zalapa JE, Cuevas H, Zhu H, Steffan S, Senalik D, Zeldin E (2012). Using next-generation sequencing approaches to isolate simple sequence repeat (SSR) loci in the plant sciences. Am J Bot.

[9] Iorizzo M, Senalik DA, Grzebelus D, Bowman M, Cavagnaro PF, Matvienko M (2011). De novo assembly and characterization of the carrot transcriptome reveals novel genes, new markers, and genetic diversity. BMC Genom.

[10] Zhang H, Wei L, Miao H, Zhang T, Wang C (2012). Development and validation of genic-SSR markers in sesame by RNA-seq. BMC Genom.

[11] Shirasawa K, Koilkonda P, Aoki K, Hirakawa H, Tabata S, Watanabe M (2012). In silico polymorphism analysis for the development of simple sequence repeat and transposon markers and construction of linkage map in cultivated peanut. BMC Plant Biol.

[12] D’Agostino N, Golas T, van de Geest H, Bombarely A, Dawood T, Zethof J (2013). Genomic analysis of the native European *Solanum* species, *S. dulcamara*. BMC Genom..

[13] Liu Z, Chen T, Ma L, Zhao Z, Zhao PX, Nan Z (2013). Global transcriptome sequencing using the Illumina platform and the development of EST-SSR markers in autotetraploid alfalfa. PLoS One.

[14] Xiao Y, Zhou L, Xia W, Mason AS, Yang Y, Ma Z (2014). Exploiting transcriptome data for the development and characterization of gene-based SSR markers related to cold tolerance in oil palm (*Elaeis guineensis*). BMC Plant Biol.

[15] Sio C-P Lu Y-L, Chen C-M, Pai T-W, Chang H-T, editors. Mining polymorphic SSRs from individual genome sequences. In: The seventh international conference on complex, intelligent, and software intensive systems (CISIS), Taichung; 2013.

[16] Hoffman JI, Nichols HJ (2011). A novel approach for mining polymorphic microsatellite markers in silico. PLoS One.

[17] Li H, Handsaker B, Wysoker A, Fennell T, Ruan J, Homer N (2009). The sequence alignment/map format and SAMtools. Bioinformatics.

[18] Gymrek M, Golan D, Rosset S, Erlich Y (2012). lobSTR: a short tandem repeat profiler for personal genomes. Genome Res.

[19] Highnam G, Franck C, Martin A, Stephens C, Puthige A, Mittelman D (2013). Accurate human microsatellite genotypes from high-throughput resequencing data using informed error profiles. Nucleic Acids Res.

[20] Fungtammasan A, Ananda G, Hile SE, Su MS, Sun C, Harris R (2015). Accurate typing of short tandem repeats from genome-wide sequencing data and its applications. Genome Res.

[21] Langmead B, Salzberg SL (2012). Fast gapped-read alignment with Bowtie 2. Nat Methods.

